# Efficacy of additional corticosteroids to multimodal cocktail periarticular injection in total knee arthroplasty: a meta-analysis of randomized controlled trials

**DOI:** 10.1186/s13018-020-02144-0

**Published:** 2021-01-22

**Authors:** Qi Li, Guo Mu, Xiangbo Liu, Milian Chen

**Affiliations:** 1 Department of Anesthesiology, Shehong People’s Hospital, NO.19, Guanghan road, Shehong, 629200 Sichuan Province People’s Republic of China; 2 Southwest Medical University, NO.319, Section 3, Zhongshan road, Jiangyang District, Luzhou, 646000 Sichuan Province People’s Republic of China; 3 Department of Anesthesiology, Zigong Fourth People’s Hospital, NO.19, Tanmulin Street, Ziliujing District, Zigong, 643000 Sichuan Province People’s Republic of China

**Keywords:** Corticosteroid, Periarticular injection, Total knee arthroplasty

## Abstract

**Background:**

As the ultimate method for the treatment of osteoarthritis, total knee arthroplasty (TKA) has been widely used in the clinic. Local injection of multimodal cocktails, including corticosteroids, is commonly used for pain management after TKA. This meta-analysis aims to systematically evaluate the effect of periarticular injection of corticosteroids on postoperative pain relief and knee functional recovery in patients undergoing TKA.

**Methods:**

PubMed, Cochrane Library, EMBASE, and Web of Science databases were comprehensively searched for all randomized controlled trials (RCTs) published before July 1, 2020, that investigated the efficacy of corticosteroids for TKA.

**Results:**

Ten RCTs involving a total of 829 patients were assessed in the meta-analysis. Compared with the control group, the visual analogue scale (VAS) score at rest of the corticosteroid group decreased significantly at postoperative day 1 (POD1), POD2, and POD3 (*p* < 0.05). Besides, the range of flexion motion of the knee joint in the corticosteroid group at POD1 and POD2 was significantly increased (*p* < 0.05); at the same time, the range of extension motion at POD2 and POD3 showed the opposite trend between the two groups (*p* < 0.05). The morphine equivalent of postoperative analgesia was significantly reduced (*p* < 0.05), and the time required for straight leg raising (SLR) was significantly shortened (*p* < 0.05). There was no significant difference between the two groups in terms of postoperative drainage, length of hospital stay, and complications such as infection, nausea, and vomiting (*p* > 0.05).

**Conclusion:**

The additional corticosteroids to multimodal cocktail periarticular injection can relieve the early pain intensity at rest after TKA, increase the early range of motion (ROM) of the knee joint, reduce the dosage of postoperative analgesics, and shorten the duration of time required for SLR. However, it has no effect on reducing postoperative complications and shortening the length of hospital stay.

**Supplementary Information:**

The online version contains supplementary material available at 10.1186/s13018-020-02144-0.

## Background

Total knee arthroplasty (TKA) is the ultimate treatment for severe knee arthritis, but it often leads to unbearable postoperative pain and pain-related knee joint dysfunction [1, 2]. Perfect postoperative analgesia is essential to improve patient comfort and promote their rapid recovery [3, 4].

Periarticular infiltration analgesia is widely used in the clinic to relieve pain after TKA [5, 6]. It can suppress the inflammation at the surgical site, provide satisfactory analgesic effects, maintain muscle strength, and reduce the consumption of opioids and related complications [6–8]. Corticosteroids have been widely used in various surgical procedures as an anti-inflammatory drug [9, 10]. Some studies have reported that the addition of corticosteroids to multimodal cocktail periarticular injection can inhibit inflammation and provide additional analgesia [11, 12], while others have found no benefit and may even increase the risk of complications [13, 14]. Therefore, in order to draw a more convincing conclusion, a comprehensive analysis of the relevant data of this kind of research has a certain clinical significance. Prior to this, some related meta-analysis has been conducted, but most of them are insufficient to a certain extent. The studies of Fan et al. [15], Zhou et al. [16], and Meng et al. [17] confused the intravenous and local application of corticosteroids. Chai et al. [18] failed to distinguish between periarticular injection and intra-articular injection in their study and did not convert different kinds of drugs in the analysis of postoperative analgesic consumption. Ultimately, the above factors led to the poor credibility of these existing meta-analyses, so it is necessary to make further analysis.

In order to solve this problem, this meta-analysis, by analyzing the current RCTs involving periarticular injection only, aims to clarify whether periarticular injection including corticosteroids can relieve postoperative pain and improve knee joint function in different time points, and explore its safety. In addition, two recent RCTs [19, 20] were included in this study to obtain a higher quality result.

## Methods

We conduct this meta-analysis according to the rules of Preferred Reporting Items for Systematic Reviews and Meta-Analyses (PRISMA) [21].

### Search strategies

The PubMed, Cochrane Library, EMBASE, and Web of Science databases were comprehensively searched for randomized controlled clinical trials (RCTs) published before July 2020 that investigated the efficacy of additional corticosteroids to multimodal cocktail periarticular injection in TKA. In addition, the reference lists of all included studies were checked for any potential additional publications. We used the keywords of knee arthroplasty, knee replacement, glucocorticoids, glucocorticosteroid, corticosteroid, and so on. The detailed search strategies for each database are presented in the Supplemental materials (Supplementary Table. 1 Details about the search strategies).

### Inclusion and exclusion criteria

Inclusion criteria:

(1) Randomized controlled trials (RCTs); (2) the target population consisted of patients undergoing unilateral primary TKA; (3) additional corticosteroids added in multimodal cocktail for the corticosteroid group; (4) no other difference between the corticosteroid group and control group besides the administration of corticosteroids; (5) only periarticular injection approach was administered; (6) the outcomes were related with VAS, range of motion (ROM) of the knee, postoperative drainage, duration of time required for straight leg raising (SLR), length of hospital stay, consumption of opioid for postoperative analgesia, and incidence of complications such as postoperative infection, wound oozing, nausea, and vomiting; (7) the full text was available.

Exclusion criteria:

(1) Patients undergoing bilateral TKA, unicondylar knee arthroplasty, or revision; (2) patients with a long history of corticosteroid medication; (3) intra-articular injection; (4) animal studies.

### Data extraction

Two reviewers independently screened the papers from their titles and abstracts and selected relevant studies that met the eligibility criteria. Data were extracted and collated independently by the same two reviewers independently with any disagreement settled by a third reviewer. We would send an e-mail to the original investigators when requisite data were lacking in the publications.

The following items were extracted: (1) basic information: name of the first author, publication date, sample size, and demographic data of participants; (2) techniques: corticosteroid type, dosages, drug regimens; perioperative medication; and anesthesia method; (3) primary outcome: VAS scores at rest and on motion and range of motion (ROM) of the knee; (4) secondary outcome: postoperative drainage, duration of time required for straight leg raising (SLR), length of hospital stay, consumption of opioid for postoperative analgesia, and incidence of complications such as postoperative infection, wound oozing, nausea, and vomiting. The raw data are presented in the supplementary materials (Supplementary Table. 2 Raw data).

### Statistical analysis

The meta-analysis was conducted using Review Manager Software (Revman 5.4, Cochrane Collaboration, Oxford, UK). Continuous data was expressed by weighted mean difference (WMD) and 95% confidence interval (CI), and dichotomous data was expressed by relative risk (RR) and 95% CI. Cochran’s *Q* test and Higgins’ *I*^2^ statistical test were used to assess the statistical heterogeneity. The results showed a low level of heterogeneity when *I*^2^ < 50%, and a fixed-effects model would be used. The results showed significant heterogeneity when *I*^2^ ≥ 50%, then sensitivity analysis and subgroup analyses would be conducted to find the source of the heterogeneity. If the heterogeneity could not be eliminated, a random effects model that estimated the uncertainty of results with sampling error and study variance would be used when the related studies had no clinical heterogeneity. Descriptive analysis was used for data that cannot be merged.

Risk of bias assessment was done by using the Cochrane Collaboration tool (Cochrane, London, UK). Finally, a funnel plot was used to assess potential publication bias visually.

## Results

### Search results and study characteristics

**Fig. 1:**
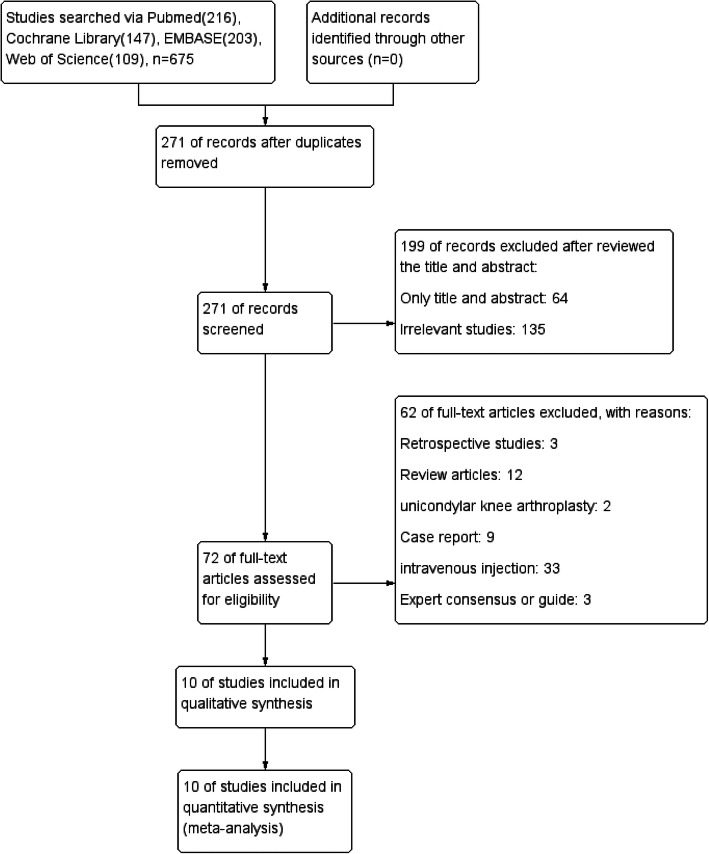
Flow chart of the study selection

A total of 675 articles were retrieved, and 271 of them remained after excluding duplicate articles. After reading the title and abstract, 199 articles were eliminated, leaving 72. Sixty-two articles were discarded for various reasons (retrospective study, review article, unicondylar knee arthroplasty, case report, intravenous injection, and expert consensus or guide) when the full text was browsed for further screening, and 10 studies [11, 12, 14, 19, 20, 22–26] finally met the inclusion criteria (Fig. 1, flow chart of study selection). The characteristics of the 10 studies which involved 829 participants are summarized in Table 1.

**Table 1:** Characteristics of the included studies

Study (author + year)	Sample size	Type of surgery	Type of anesthesia	Basic information				Cocktail	Corticosteroids	Postoperative analgesia
				Age	Male/female	BMI	Corticosteroids/control group			
Kwon 2014 [24]	152	TKA	SA	69.3/69.3	0/76 0/76	25.9/25.9	76/76	Morphine 10 mg, ropivacaine 300 mg, ketorolac 30 mg, 1:1000 epinephrine, 300 μg	Triamcinolone 40 mg	NM
Yue 2013 [26]	72	TKA	GA	70.2 ± 6.4/69.3 ± 5.7	32/4 32/4	25.23 ± 4.81/26.14 ± 3.27	36/36	0.75% ropivacaine 30 mL, 1:1000 adrenaline 0.5 mL	Betamethasone 7 mg	PCA (morphine)
Ikeuchi 2014 [12]	40	TKA	GA	77 ± 6/76 ± 3	2/18 4/16	NM	20/20	0.75% ropivacaine 20 mL, isepamicin 400 mg	Dexamethasone 6.6 mg	PCA (fentanyl)
Chia 2013 [14]	85	TKA	SA	66.8 ± 7.5/65.1 ± 8.4	NM	32.09 ± 5.4/31.49 ± 4.7	42/43	0.2% ropivacaine 100 mL, 1:1000 adrenaline	Triamcinolone 80 mg	PCA (morphine, no specific data)
Sean 2011 [25]	100	TKA	GA21 + SA29/GA20 + SA30	67.9/65.4	NM	26.7/27.3	50/50	1:200,000 epinephrine and 0.5% bupivacaine 0.5 mL/kg	Triamcinolone 40 mg	PCA (morphine)
Christensen 2009 [22]	76	TKA	GA	65.8 ± 1.1/65.2 ± 11	16/23 7/30	32.9 ± 6.5/35.1 ± 8	39/37	Bupivacaine 80 mg, morphine 4 mg, epinephrine 300 mg, clonidine 100 mg, cefuroxime 750 mg	Methylprednisolone 40 mg	PCA (morphine)
Kim 2015 [23]	86	TKA	SA	71.4 ± 4.7/70.6 ± 5.5	2/41 4/39	25.8 ± 3.3/27.1 ± 4.0	43/43	Ropivacaine 180 mg, morphine sulfate 5 mg, ketorolac 30 mg, cefazolin 1 g, 1:1000 epinephrine 0.6 mL	Methylprednisolone 40 mg	PCA (no specific data)
Iseki 2019 [20]	41	TKA	SA	72 ± 7/76 ± 8	2/19 5/15	26.4 ± 4.1/26.3 ± 3.7	21/20	Ropivacaine (7.5 mg/mL) 40 mL, morphine hydrochloride hydrate (10 mg/mL) 0.8 mL, epinephrine (1.0 mg/mL) 0.3 mL, fketoprofen 50 mg	Methylprednisolone 40 mg	Diclofenac sodium suppository
Tsukada 2016 [11]	75	TKA	SA	75/72	3/35 5/32	26.7/27.3	38/37	Ropivacaine (7.5 mg/mL) 40 mL, morphine hydrochloride hydrate (10 mg/mL) 0.8 mL, epinephrine (1.0 mg/mL) 0.3 mL, ketoprofen 50 mg	Methylprednisolone 40 mg	Diclofenac sodium suppository (no specific data)
Wang 2020 [19]	102	TKA	GA	65.1 ± 8.6/63.9 ± 6.4	17/35 15/35	26 ± 2.7/26.1 ± 3.5	52/50	0.2% ropivacaine, epinephrine (2.0 μg/mL), 100 mL	Dexamethasone 10 mg	PCA (morphine)

*SA* spinal anesthesia, *GA* general anesthesia, *TKA* total knee arthroplasty, *PCA* patient-controlled analgesia, *NM* not mentioned

### Primary outcomes

#### VAS score at rest

**Fig. 2:**
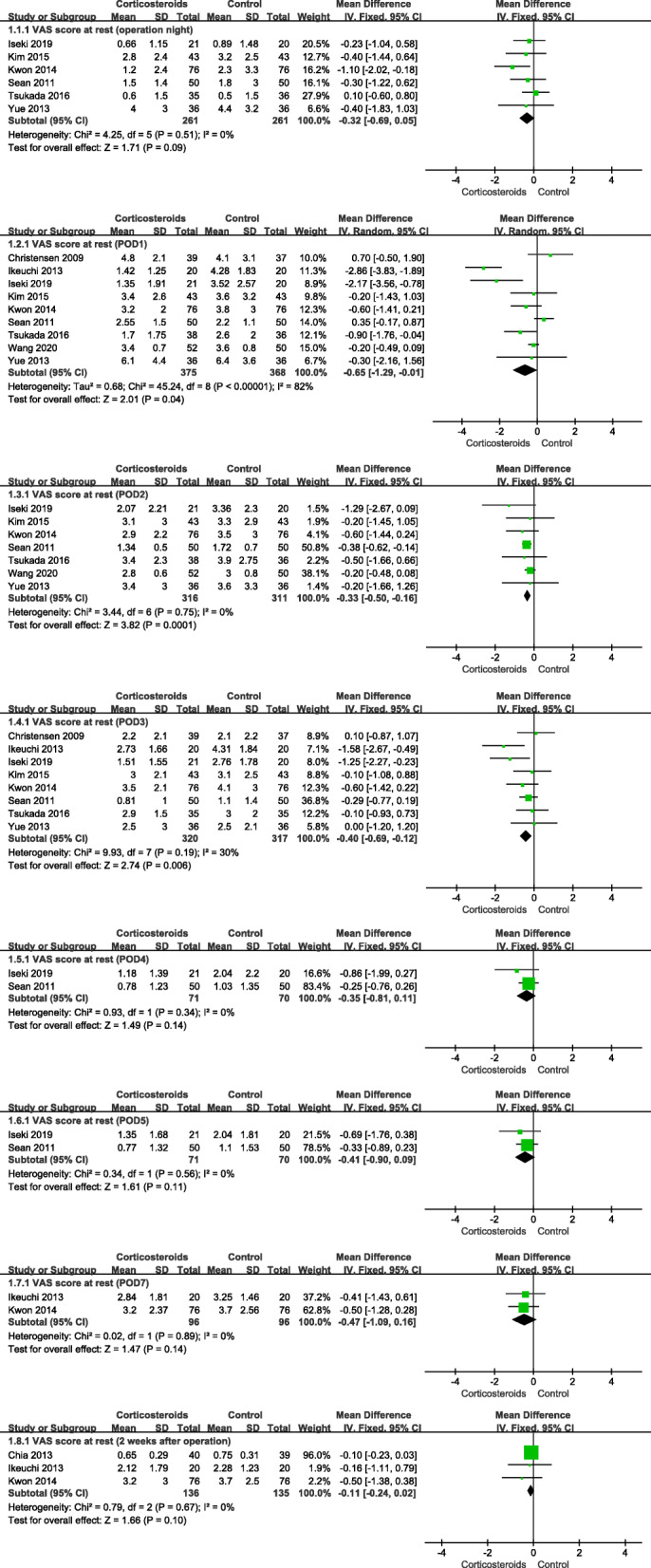
Forest plot showing VAS score at rest at POD1, POD2, POD3, POD4, POD5, POD7, and 2 weeks after the operation

The pooling results showed that the VAS score at rest of the corticosteroid group was significantly lower than that of the control group at postoperative day 1 (POD1) (MD − 0.65; 95% CI − 1.29 to − 0.01; *p* < 0.05; *I*^2^ = 82%), POD2 (MD − 0.33; 95% CI − 0.50 to − 0.16; *p* < 0.05; *I*^2^ = 0%), and POD3 (MD − 0.40; 95% CI − 0.69 to − 0.12; *p* < 0.05; *I*^2^ = 30%) day after the operation. There was no significant difference in VAS score at the operation night, POD4, POD5, POD7, and 2 weeks after the operation. The meta-analysis result at POD1 showed heterogeneity. Sensitivity analysis and subgroup analysis failed to find the source of heterogeneity. Considering that there was no clinical heterogeneity in these studies, a random effect model was used to analyze it. The rest of the data was analyzed using fixed effects (Fig. 2, VAS score at rest).

#### VAS score on motion

**Fig. 3:**
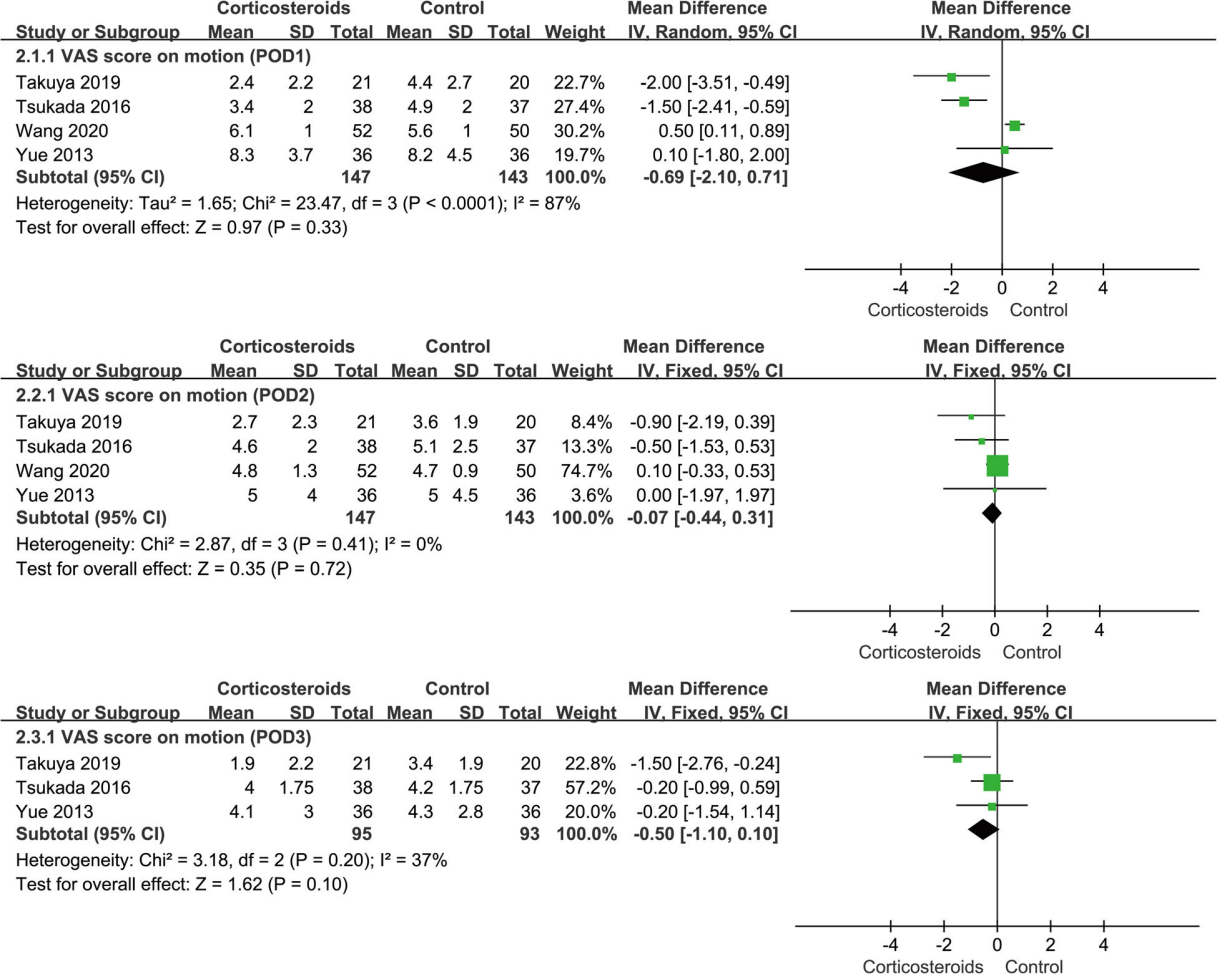
Forest plot showing VAS score on motion at POD1, POD2, and POD3

The results showed that there was no significant difference in VAS score on motion between the corticosteroid group and control group at POD1, POD2, and POD3. The meta-analysis results showed obvious heterogeneity at POD1. Sensitivity analysis and subgroup analysis could not eliminate heterogeneity, so a random effect model was used to analyze the relevant results. The rest of the data were analyzed by fixed effect. VAS scores at other time points after operation were not analyzed because of the limited sample size (Fig. 3, VAS score on motion).

#### Range of flexion motion

**Fig. 4:**
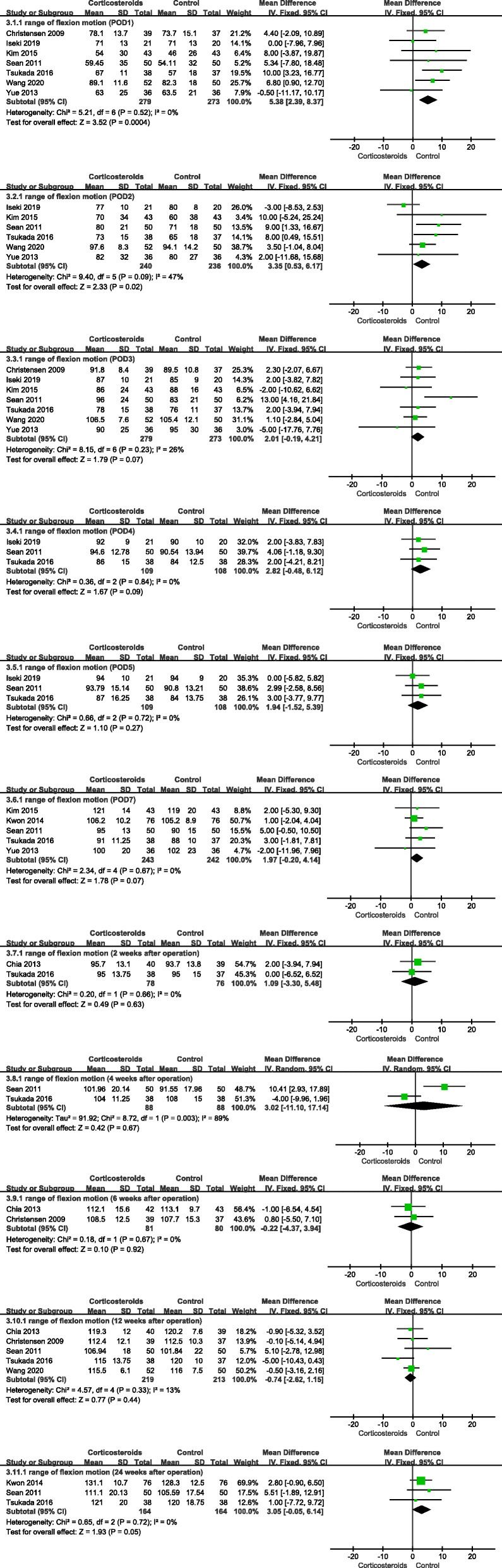
Forest plot showing range of flexion motion at POD1, POD2, POD3, POD4, POD5, POD7, and 2, 4, 6, 12, and 24 weeks after the operation

Compared with the control group, the range of flexion motion of the knee joint was increased in the corticosteroid group at POD1 (MD 5.38; 95% CI 2.39 to 8.37; *p* < 0.05; *I*^2^ = 0%) and POD2 (MD 3.35; 95% CI 0.53 to 6.17; *p* < 0.05; *I*^2^ = 47%). However, there was no significant difference between the two groups at POD3, POD4, POD5, POD7, and 2, 4, 6, 12, and 24 weeks after surgery. Heterogeneity was detected at 4 weeks after the operation. Sensitivity analysis and subgroup analysis failed to eliminate the heterogeneity, so the random effect model was used (Fig. 4, range of flexion motion).

#### Range of extension motion

**Fig. 5:**
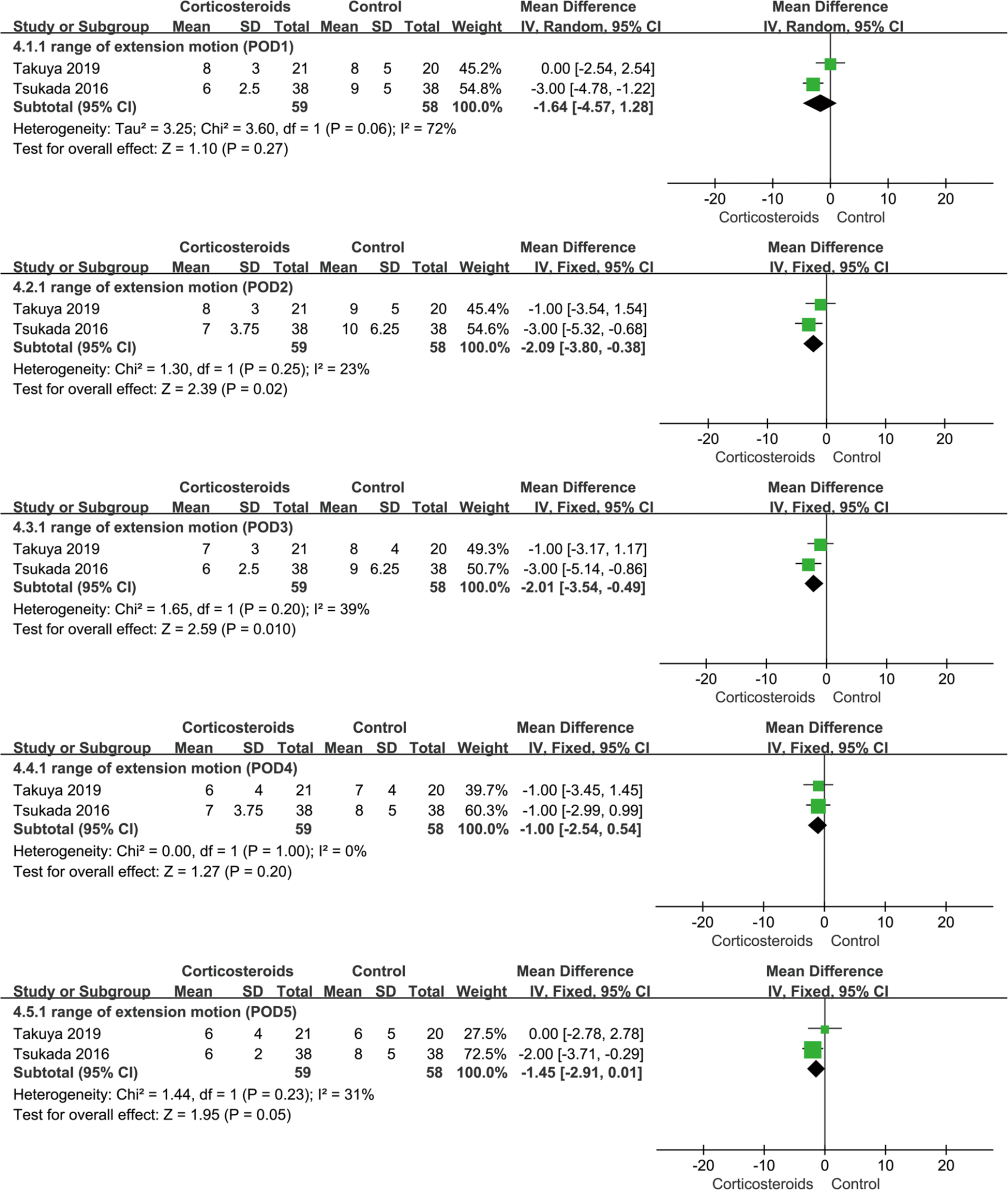
Forest plot showing range of extension motion at POD1, POD2, POD3, POD4, and POD5

The results showed that the range of extension motion in the corticosteroid group decreased at POD2 (MD − 2.09; 95% CI − 3.80 to − 0.38; *p* < 0.05; *I*^2^ = 23%) and POD3 (MD − 2.01; 95% CI − 3.54 to − 0.49; *p* < 0.05; *I*^2^ = 39%) compared with the control group. On the contrary, there was no significant difference between the two groups at POD1, POD4, and POD5. There was certain heterogeneity in the data at POD1, but it could not be eliminated after sensitivity analysis and subgroup analysis. After confirming that there was no clinical heterogeneity in the related studies, the random effect model was applied (Fig. 5, range of extension motion).

### Secondary outcomes

#### Morphine equivalent for postoperative analgesia

**Fig. 6:**
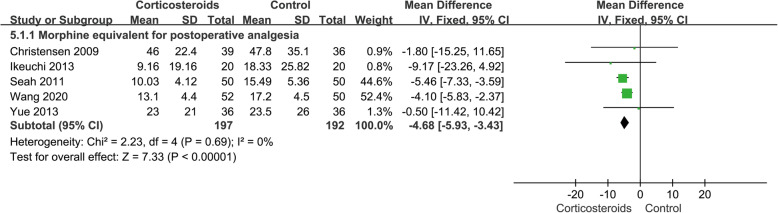
Forest plot showing morphine equivalent for postoperative analgesia

A total of 5 studies, 389 cases, involved opioid consumption for postoperative analgesia. All opioid doses were converted to corresponding morphine equivalent for statistical analysis in this meta-analysis. The results showed that the postoperative analgesic dosage of patients in the corticosteroid group was significantly less than that of the control group (MD − 4.68; 95% CI − 5.93 to − 3.43; *p* < 0.05; *I*^2^ = 0%) (Fig. 6, morphine equivalent).

#### Postoperative drainage

**Fig. 7:**
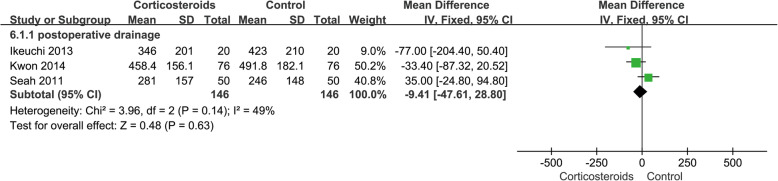
Forest plot showing postoperative drainage

The postoperative knee joint drainage was recorded in 3 studies including 292 patients. The results showed that there was no significant difference in drainage volume between the two groups (MD − 9.41; 95% CI − 47.61 to 28.80; *p* > 0.05; *I*^2^ = 49%) (Fig. 7, postoperative drainage).

#### Time required for straight leg raising (SLR) and length of hospital stay

**Fig. 8:**
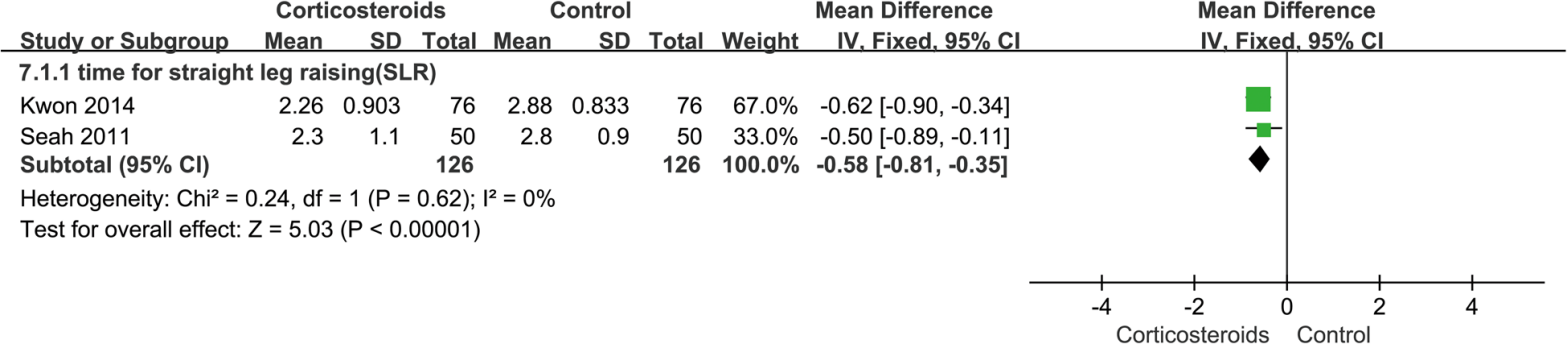
Forest plot showing time for straight leg raising

**Fig. 9:**
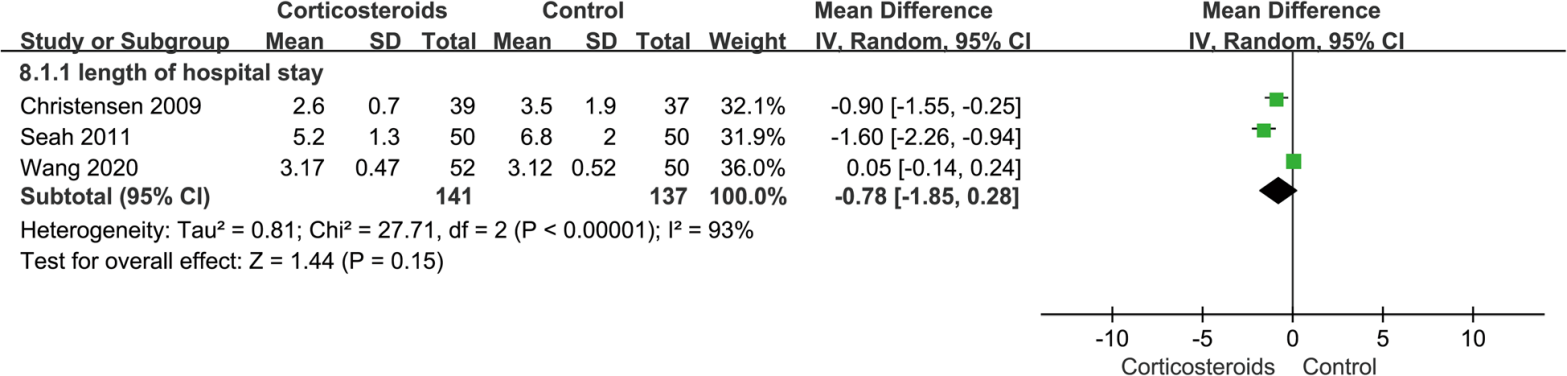
Forest plot showing length of hospital stay

The time required for SLR was shown in 2 studies, involving 252 cases, and better outcome was shown in the corticosteroid group (MD − 0.58; 95% CI − 0.81 to − 0.35; *p* < 0.05; *I*^2^ = 0%) (Fig. 8, time required for straight leg raising). The length of hospital stay was reported in 3 studies, with 278 cases, and the pooled outcomes showed no significant difference (MD − 0.78; 95% CI − 1.85 to 0.28; *p* > 0.05; *I*^2^ = 93%) (Fig. 9, length of hospital stay). The random effect model was used to analyze data about the length of hospital stay, because related studies had clinical homogeneity; besides, sensitivity analysis and subgroup analysis could not eliminate the heterogeneity.

#### Incidence of complications

**Fig. 10:**
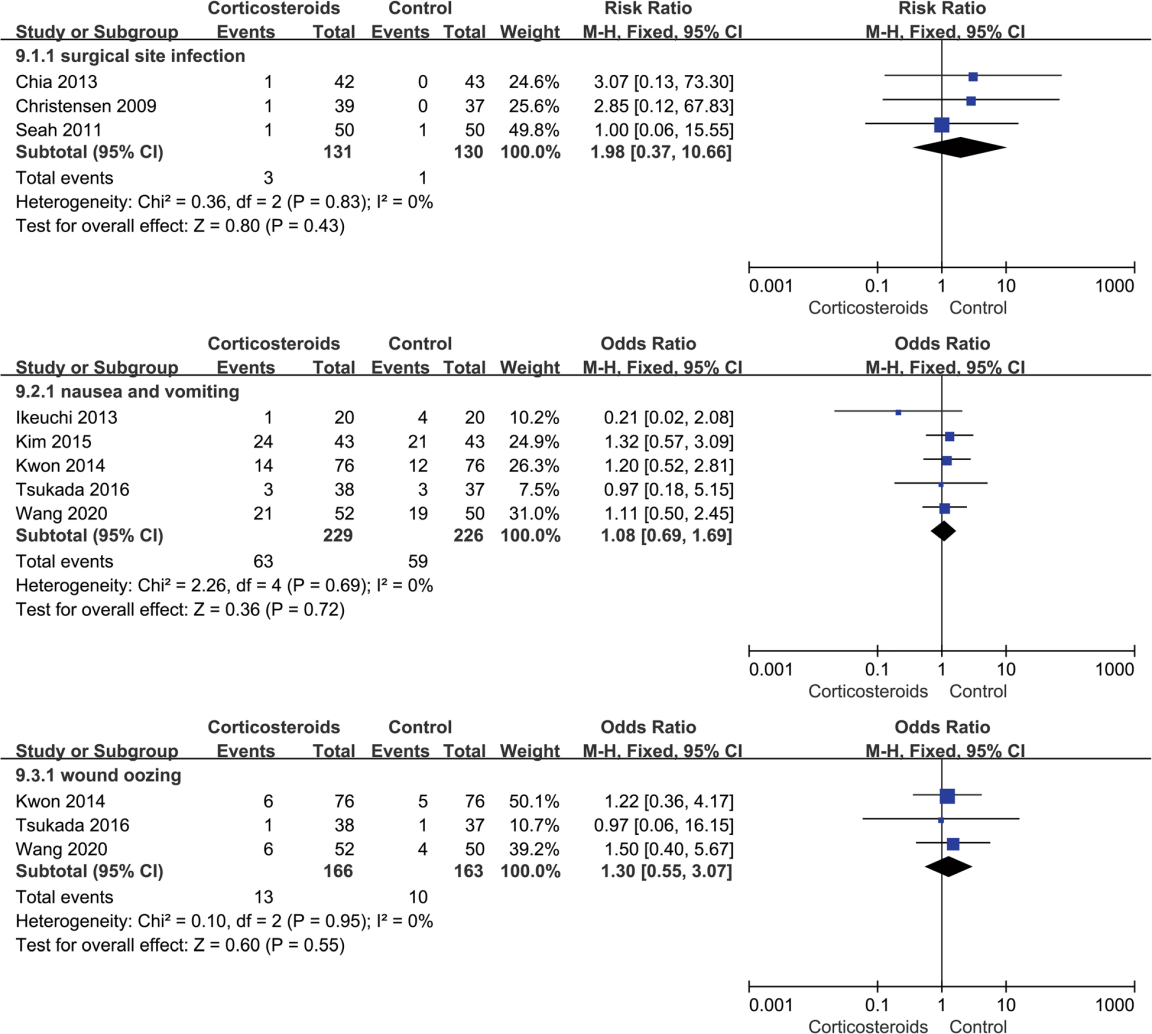
Forest plot showing incidence of complications

A total of 9 studies, 758 cases, reported the occurrence of postoperative surgical site infection. Six of the studies showed that there was no postoperative infection that occurred in the two groups. The meta-analysis results of the remaining three studies indicated that no significant difference existed in the incidence of infection between the two groups (RR 1.98; 95% CI 0.37 to 10.66; *p* > 0.05; *I*^2^ = 0%). Two studies involving 202 cases reported no tendon rupture occurred in both groups. Five studies, involving 455 cases, recorded postoperative nausea and vomiting (RR 1.08; 95% CI 0.69 to 1.69; *p* > 0.05; *I*^2^ = 0%), and 3 studies with 329 cases showed the wound oozing (RR 1.30; 95% CI 0.55 to 3.07; *p* > 0.05; *I*^2^ = 0%). Pooled outcomes showed no significant difference (Fig. 10, incidence of complications).

### Bias assessment

**Fig. 11:**
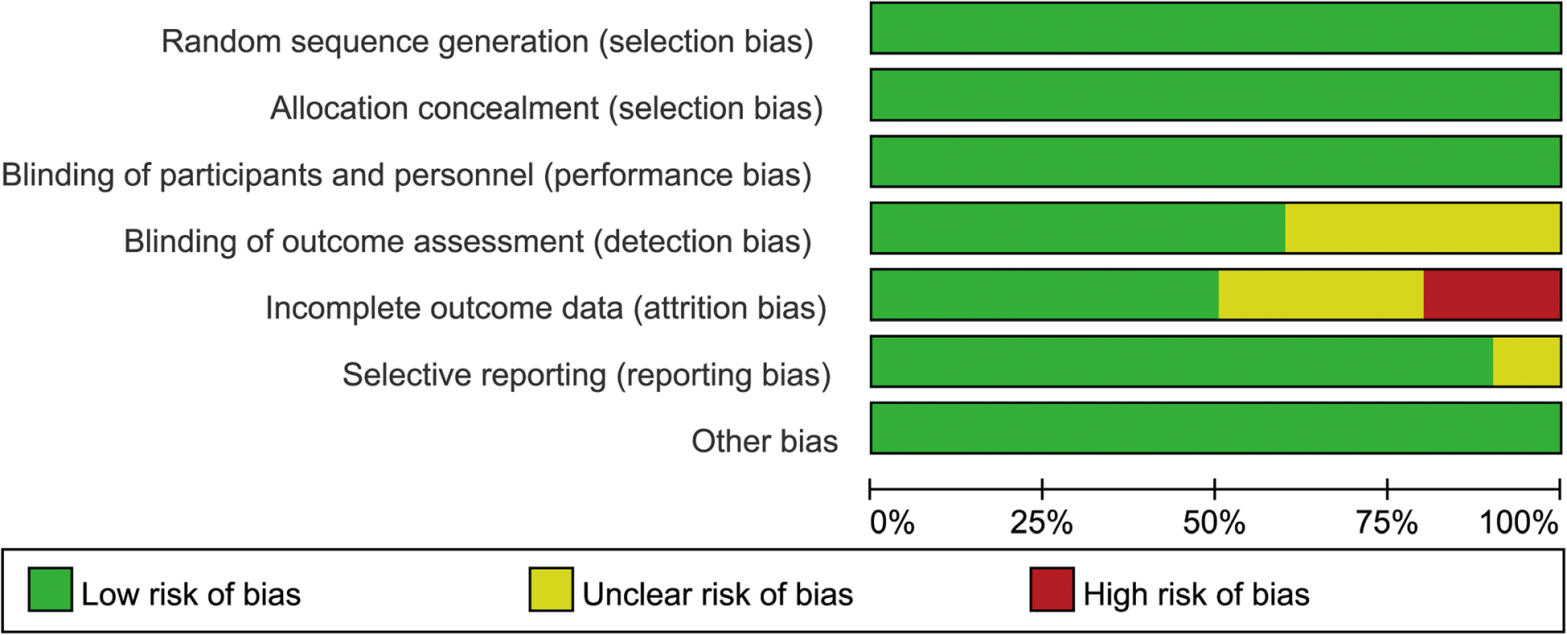
Risk of bias

**Fig. 12:**
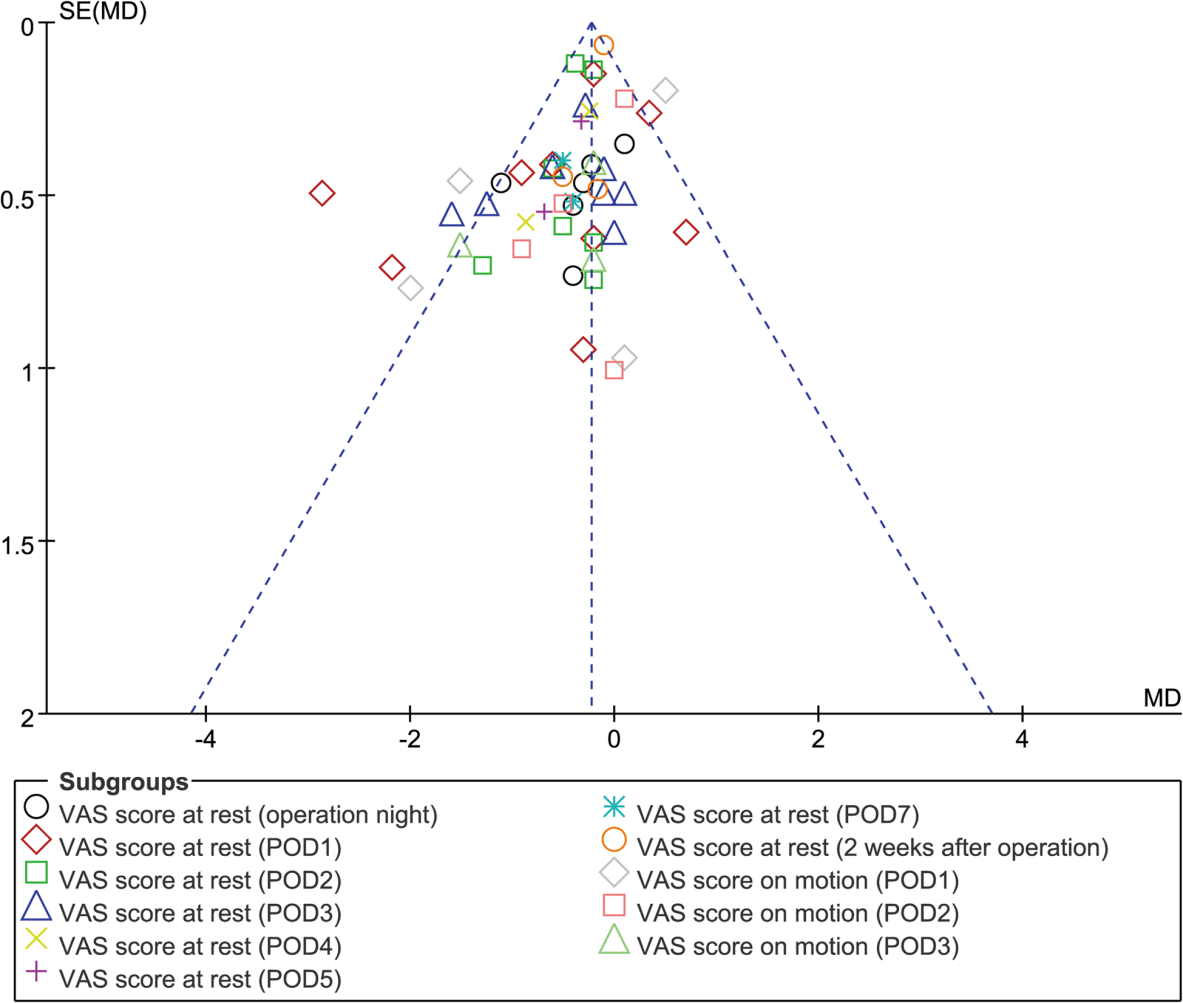
Funnel plot

We can learn from the risk of bias graph (Fig. 11, risk of bias graph) that two studies [14, 23] had a high risk for attrition bias, while other studies have not found any high-risk items. As for the publication bias, the funnel plot has no obvious asymmetry, so we concluded that there was no significant publication bias (Fig. 12, funnel plot).

## Discussion

Severe postoperative pain of TKA significantly affects the patient’s experience and postoperative rapid recovery. Exploring effective postoperative analgesia is of great significance. Patient-controlled intravenous analgesia (PCIA) often requires large doses of opioid analgesics, which often lead to complications such as nausea and vomiting, skin itching, and even respiratory depression [27]. Epidural block and femoral nerve block are effective methods for pain control after TKA, but they may weaken the strength of quadriceps femoris and affect the early rehabilitation of patients [6, 28, 29]. Adductor canal block can provide the same postoperative analgesic effect as femoral nerve block without inhibiting the strength of quadriceps femoris, but it cannot effectively cover the lateral and posterior part of the knee [30, 31]. From the above, it can be seen that conventional analgesic methods are difficult to provide perfect analgesic effect after TKA, and it is of great clinical value to explore new analgesic methods. Some studies have shown that periarticular injection can obtain good analgesic effect and even improve the postoperative range of motion and promote rehabilitation [6, 32, 33]. Local injections often contain local anesthetics such as ropivacaine or bupivacaine, epinephrine, nonsteroidal anti-inflammatory drugs, and morphine [6, 22, 34]. In addition, some studies have added corticosteroids to multimodal cocktail periarticular injection, but the results are not consistent [12, 14, 22, 24–26, 35]. Although current studies have not found that corticosteroids increase the risk of serious complications such as postoperative infection, their clinical application is still concerned. The purpose of this meta-analysis was to evaluate whether the addition of corticosteroids to periarticular injection can relieve postoperative pain and promote knee function recovery after TKA, and to determine whether it is safe enough.

The results of this study showed that the addition of corticosteroids to the periarticular injection could relieve the pain score at rest from POD1 to POD3 and improve flexion range of POD1 and POD2 and extension range of POD2 and POD3. In addition, it also reduces the postoperative consumption of opioids and promotes the recovery of straight leg raising function. Similar to previous studies, corticosteroids did not increase the incidence of adverse reactions such as infection, tendon rupture, wound oozing, nausea, and vomiting. However, our research results show that corticosteroids are not significantly helpful in relieving pain scores on motion, reducing wound drainage, and shortening hospital stay.

There is a close relationship between postoperative pain and inflammatory response [36]. Some studies have shown that corticosteroids can alleviate pain by inhibiting inflammatory response [19, 37]. However, due to the lack of relevant studies, this meta-analysis did not make detailed statistics on inflammatory indicators such as C-reactive protein, erythrocyte sedimentation rate, and IL-6. Better knee function recovery may be related to better pain relief. There was no significant difference in the range of knee joint motion between the two groups at POD4, which may reflect that corticosteroids cannot provide long-lasting analgesia. In summary, our results show that although the addition of corticosteroids to multimodal cocktail periarticular injection does not significantly help the long-term rehabilitation after TKA, it can obviously alleviate the early postoperative pain and improve the early knee joint function, and it did not increase the incidence of related complications.

The limitations of our study mainly include the following aspects. (1) There were only 10 randomized controlled trials and 827 cases in our study. Therefore, a larger sample size is needed for further research, and it is even possible to compare and analyze the efficacy of different corticosteroids. (2) Meta-analysis of some ROM, VAS score, and length of hospital stay showed heterogeneity, but sensitivity analysis and subgroup analysis failed to eliminate it. After excluding clinical heterogeneity, the random effects model is used for data processing, which may have a slight impact on the reliability of the result. (3) The included studies lack inflammatory indicators such as IL-6, C-reactive protein, and erythrocyte sedimentation rate, which cannot explain the related mechanism in a deeper level.

## Conclusion

Adding corticosteroids into periarticular injection can relieve pain score at rest and improve the ROM of the knee in early stage after TKA. In addition, it can reduce the consumption of opioids and promote the recovery of straight leg raising function. Moreover, the use of corticosteroids did not increase the incidence of adverse reactions such as infection, tendon rupture, wound ozzing, nausea, and vomiting. However, it had no significant effect on relieving pain scores on motion, reducing wound drainage, and shortening hospital stay.

## Supplementary Information

**Additional file 1: Supplementary Table 1.** Details about the search strategies.

**Additional file 2: Supplementary Table 2.** Raw data.

## Data Availability

All data generated or analyzed during this study are included in published articles.

## Abbreviations

TKA: Total knee arthroplasty
RCTs: Randomized controlled trials
VAS: Visual analogue scale
POD1: Postoperative day 1
ROM: Range of motion
SLR: Straight leg raising
PRISMA: Preferred Reporting Items for Systematic Reviews and Meta-Analyses
WMD: Weighted mean difference
CI: Confidence interval
RR: Relative risk

## References

[1] Skou ST, Roos EM, Laursen MB, Rathleff MS, Arendt-Nielsen L, Simonsen O (2015). A randomized, controlled trial of total knee replacement. N Engl J Med..

[2] Parvataneni HK, Shah VP, Howard H, Cole N, Ranawat AS, Ranawat CS (2007). Controlling pain after total hip and knee arthroplasty using a multimodal protocol with local periarticular injections: a prospective randomized study. J Arthroplasty..

[3] Auyong DB, Allen CJ, Pahang JA, Clabeaux JJ, MacDonald KM, Hanson NA (2015). Reduced length of hospitalization in primary total knee arthroplasty patients using an updated enhanced recovery after orthopedic surgery (ERAS) pathway. J Arthroplasty..

[4] Khan SK, Malviya A, Muller SD, Carluke I, Partington PF, Emmerson KP (2014). Reduced short-term complications and mortality following enhanced recovery primary hip and knee arthroplasty: results from 6,000 consecutive procedures. Acta Orthop..

[5] Kurosaka K, Tsukada S, Seino D, Morooka T, Nakayama H, Yoshiya S (2016). Local infiltration analgesia versus continuous femoral nerve block in pain relief after total knee arthroplasty: a randomized controlled trial. J Arthroplasty..

[6] Li D, Tan Z, Kang P, Shen B, Pei F (2017). Effects of multi-site infiltration analgesia on pain management and early rehabilitation compared with femoral nerve or adductor canal block for patients undergoing total knee arthroplasty: a prospective randomized controlled trial. Int Orthop..

[7] Pepper AM, Mercuri JJ, Behery OA, Vigdorchik JM (2018). Total hip and knee arthroplasty perioperative pain management: what should be in the cocktail. JBJS Rev..

[8] Motififard M, Omidian A, Badiei S (2017). Pre-emptive injection of peri-articular-multimodal drug for post-operative pain management in total knee arthroplasty: a double-blind randomized clinical trial. Int Orthop..

[9] Vayne-Bossert P, Haywood A, Good P, Khan S, Rickett K, Hardy JR (2017). Corticosteroids for adult patients with advanced cancer who have nausea and vomiting (not related to chemotherapy, radiotherapy, or surgery). Cochrane Database Syst Rev..

[10] DREAMS Trial Collaborators and West Midlands Research Collaborative (2017). Dexamethasone versus standard treatment for postoperative nausea and vomiting in gastrointestinal surgery: randomised controlled trial (DREAMS Trial). BMJ.

[11] Tsukada S, Wakui M, Hoshino A (2016). The impact of including corticosteroid in a periarticular injection for pain control after total knee arthroplasty: a double-blind randomised controlled trial. Bone Joint J..

[12] Ikeuchi M, Kamimoto Y, Izumi M, Fukunaga K, Aso K, Sugimura N (2014). Effects of dexamethasone on local infiltration analgesia in total knee arthroplasty: a randomized controlled trial. Knee Surg Sports Traumatol Arthrosc..

[13] Ali A, Sundberg M, Hansson U, Malmvik J, Flivik G (2015). Doubtful effect of continuous intraarticular analgesia after total knee arthroplasty: a randomized double-blind study of 200 patients. Acta Orthop..

[14] Chia SK, Wernecke GC, Harris IA, Bohm MT, Chen DB, Macdessi SJ (2013). Peri-articular steroid injection in total knee arthroplasty: a prospective, double blinded, randomized controlled trial. J Arthroplasty..

[15] Fan Z, Ma J, Kuang M, Zhang L, Han B, Yang B (2018). The efficacy of dexamethasone reducing postoperative pain and emesis after total knee arthroplasty: a systematic review and meta-analysis. Int J Surg..

[16] Zhou G, Ma L, Jing J, Jiang H (2018). A meta-analysis of dexamethasone for pain management in patients with total knee arthroplasty. Medicine (Baltimore).

[17] Meng J, Li L (2017). The efficiency and safety of dexamethasone for pain control in total joint arthroplasty: a meta-analysis of randomized controlled trials. Medicine (Baltimore)..

[18] Chai X, Liu H, You C, Wang C (2019). Efficacy of additional corticosteroid in a multimodal cocktail for postoperative analgesia following total knee arthroplasty: a meta-analysis of randomized controlled trials. Pain Pract..

[19] Wang Q, Tan G, Mohammed A, Zhang Y, Li D, Chen L, et al. Adding corticosteroids to periarticular infiltration analgesia improves the short-term analgesic effects after total knee arthroplasty: a prospective, double-blind, randomized controlled trial. Knee Surg Sports Traumatol Arthrosc. 2020. 10.1007/s00167-020-06039-9 32361928

[20] Iseki T, Tsukada S, Wakui M, Kurosaka K, Yoshiya S (2019). Percutaneous periarticular multi-drug injection at one day after total knee arthroplasty as a component of multimodal pain management: a randomized control trial. BMC Musculoskelet Disord..

[21] Moher D, Liberati A, Tetzlaff J, Altman DG, PRISMA Group (2009). Preferred reporting items for systematic reviews and meta-analyses: the PRISMA statement. BMJ..

[22] Christensen CP, Jacobs CA, Jennings HR (2009). Effect of periarticular corticosteroid injections during total knee arthroplasty. A double-blind randomized trial. J Bone Joint Surg Am..

[23] Kim TW, Park SJ, Lim SH, Seong SC, Lee S, Lee MC (2015). Which analgesic mixture is appropriate for periarticular injection after total knee arthroplasty? Prospective, randomized, double-blind study. Knee Surg Sports Traumatol Arthrosc..

[24] Kwon SK, Yang IH, Bai SJ, Han CD (2014). Periarticular injection with corticosteroid has an additional pain management effect in total knee arthroplasty. Yonsei Med J..

[25] Sean VW, Chin PL, Chia SL, Yang KY, Lo NN, Yeo SJ (2011). Single-dose periarticular steroid infiltration for pain management in total knee arthroplasty: a prospective, double-blind, randomised controlled trial. Singapore Med J..

[26] Yue DB, Wang BL, Liu KP, Guo WS (2013). Efficacy of multimodal cocktail periarticular injection with or without steroid in total knee arthroplasty. Chin Med J (Engl).

[27] Vendittoli PA, Makinen P, Drolet P, Lavigne M, Fallaha M, Guertin MC (2006). A multimodal analgesia protocol for total knee arthroplasty. A randomized, controlled study. J Bone Joint Surg Am..

[28] Li D, Yang Z, Xie X, Zhao J, Kang P (2016). Adductor canal block provides better performance after total knee arthroplasty compared with femoral nerve block: a systematic review and meta-analysis. Int Orthop..

[29] Grevstad U, Mathiesen O, Valentiner LS, Jaeger P, Hilsted KL, Dahl JB (2015). Effect of adductor canal block versus femoral nerve block on quadriceps strength, mobilization, and pain after total knee arthroplasty: a randomized, blinded study. Reg Anesth Pain Med..

[30] Li D, Ma GG (2016). Analgesic efficacy and quadriceps strength of adductor canal block versus femoral nerve block following total knee arthroplasty. Knee Surg Sports Traumatol Arthrosc..

[31] Sogbein OA, Sondekoppam RV, Bryant D, Johnston DF, Vasarhelyi EM, MacDonald S (2017). Ultrasound-guided motor-sparing knee blocks for postoperative analgesia following total knee arthroplasty: a randomized blinded study. J Bone Joint Surg Am..

[32] Barrington JW, Lovald ST, Ong KL, Watson HN, Emerson RH (2016). Postoperative pain after primary total knee arthroplasty: comparison of local injection analgesic cocktails and the role of demographic and surgical factors. J Arthroplasty..

[33] Gwam CU, Mistry JB, Richards IV, Patel D, Patel NG, Thomas M (2018). Does addition of adductor canal blockade to multimodal periarticular analgesia improve discharge status, pain levels, opioid use, and length of stay after total knee arthroplasty. J Knee Surg..

[34] Ritter MA, Koehler M, Keating EM, Faris PM, Meding JB (1999). Intra-articular morphine and/or bupivacaine after total knee replacement. J Bone Joint Surg Br..

[35] Pang HN, Lo NN, Yang KY, Chong HC, Yeo SJ (2008). Peri-articular steroid injection improves the outcome after unicondylar knee replacement: a prospective, randomised controlled trial with a two-year follow-up. J Bone Joint Surg Br..

[36] Riegler FX. Update on perioperative pain management. Clin Orthop Relat Res. 1994:283–92. 8050240

[37] Creamer P (1997). Intra-articular corticosteroid injections in osteoarthritis: do they work and if so, how. Ann Rheum Dis..

