# Correction to: Efficacy of additional corticosteroids to multimodal cocktail periarticular injection in total knee arthroplasty: a meta-analysis of randomized controlled trials

**DOI:** 10.1186/s13018-021-02317-5

**Published:** 2021-03-19

**Authors:** Qi Li, Guo Mu, Xiangbo Liu, Milian Chen

**Affiliations:** 1 Department of Anesthesiology, Shehong People’s Hospital, NO.19, Guanghan road, Shehong, 629200 Sichuan Province People’s Republic of China; 2 Department of Anesthesiology, Zigong Fourth People’s Hospital, NO.19 Tanmulin Street, Ziliujin, Zigong, 643000 Sichuan Province People’s Republic of China; 3 Southwest Medical University, NO.319, Section 3, Zhongshan road, Jiangyang District, Luzhou, 643000 Sichuan Province People’s Republic of China

**Correction to: J Orthop Surg Res 16, 77 (2021)**

**https://doi.org/10.1186/s13018-020-02144-0**

Following the publication of the original article [1], the authors found out mistake on the affiliation section. They did not find this error during proofreading. The authors would like to apologize for this mistake.

The order of all authors remains the same, but the error lies on affiliations 2 and 3. The correct affiliations are shown below. These have also been updated in this article.

2 Department of Anesthesiology, Zigong Fourth People’s Hospital, NO.19 Tanmulin Street, Ziliujin, Zigong 643000, Sichuan Province, People’s Republic of China.

3 Southwest Medical University, NO.319, Section 3, Zhongshan road, Jiangyang District, Luzhou 643000, Sichuan Province, People’s Republic of China.
