# Supplementary material for: Efficacy of additional corticosteroids to multimodal cocktail periarticular injection in total knee arthroplasty: a meta-analysis of randomized controlled trials
Source: J Orthop Surg Res. 2021 Jan 22;16:77. doi: 10.1186/s13018-020-02144-0 (PMC7821531; doi:10.1186/s13018-020-02144-0)
Supplement: Supplementary file 1 — Additional file 1: Supplementary Table 1. Details about the search strategies. [file 13018_2020_2144_MOESM1_ESM.docx]

Supplementary Table 1 Details about the search strategies

| Search strategy terms for PubMed, Cochrane Library, EMBASE and Web of Science databases. |
| --- |
| The PubMed search terms were as follows:216  ID Search  #1 ((((("Glucocorticoids"[Mesh]) OR (Glucocorticoid[Title/Abstract])) OR (Glucocorticoid Effect[Title/Abstract])) OR (Effect, Glucocorticoid[Title/Abstract])) OR (Glucorticoid Effects[Title/Abstract])) OR (Effects, Glucorticoid[Title/Abstract])  #2 (((((((("Hydrocortisone"[Mesh]) OR (Cortisol[Title/Abstract])) OR (Hydrocortisone, (9 beta,10 alpha,11 alpha)-Isomer[Title/Abstract])) OR (Hydrocortisone, (11 alpha)-Isomer[Title/Abstract])) OR (11-Epicortisol[Title/Abstract])) OR (11 Epicortisol[Title/Abstract])) OR (Epicortisol[Title/Abstract])) OR (Cortifair[Title/Abstract])) OR (Cortril[Title/Abstract])  #3 (((("Cortisone"[Mesh]) OR (Cortisone Acetate[Title/Abstract])) OR (17-Hydroxy-3,11,20-trioxopregn-4-en-21-yl acetate[Title/Abstract])) OR (Cortone Acetate[Title/Abstract])) OR (Adreson[Title/Abstract])  #4 (((((((((((((((((((((((((((((((("Prednisone"[Mesh]) OR (DehydrocDehydrocortisoneortisone[Title/Abstract])) OR (delta-Cortisone[Title/Abstract])) OR (Rectodelt[Title/Abstract])) OR (Prednison Hexal[Title/Abstract])) OR (Sterapred[Title/Abstract])) OR (Ultracorten[Title/Abstract])) OR (Winpred[Title/Abstract])) OR (Apo-Prednisone[Title/Abstract])) OR (Cortan[Title/Abstract])) OR (Cortancyl[Title/Abstract])) OR (Panafcort[Title/Abstract])) OR (Cutason[Title/Abstract])) OR (Decortin[Title/Abstract])) OR (Dacortin[Title/Abstract])) OR (Decortisyl[Title/Abstract])) OR (Deltasone[Title/Abstract])) OR (Encortone[Title/Abstract])) OR (Encorton[Title/Abstract])) OR (Enkortolon[Title/Abstract])) OR (Kortancyl[Title/Abstract])) OR (Liquid Pred[Title/Abstract])) OR (Meticorten[Title/Abstract])) OR (Orasone[Title/Abstract])) OR (Panasol[Title/Abstract])) OR (Predni Tablinen[Title/Abstract])) OR (Prednidib[Title/Abstract])) OR (Predniment[Title/Abstract])) OR (Prednison Acsis[Title/Abstract])) OR (Acsis, Prednison[Title/Abstract])) OR (Pronisone[Title/Abstract])) OR (Sone[Title/Abstract])) OR (Prednison Galen[Title/Abstract])  #5 (((("Prednisolone"[Mesh]) OR (Predate[Title/Abstract])) OR (Predonine[Title/Abstract])) OR (Di-Adreson-F[Title/Abstract])) OR (Di Adreson F[Title/Abstract])  #6 (("Triamcinolone"[Mesh]) OR (Volon[Title/Abstract])) OR (Aristocort[Title/Abstract])  #7 ((((((((((((("Dexamethasone"[Mesh]) OR (Methylfluorprednisolone[Title/Abstract])) OR (Hexadecadrol[Title/Abstract])) OR (Decameth[Title/Abstract])) OR (Decaspray[Title/Abstract])) OR (Dexasone[Title/Abstract])) OR (Dexpak[Title/Abstract])) OR (Maxidex[Title/Abstract])) OR (Millicorten[Title/Abstract])) OR (Oradexon[Title/Abstract])) OR (Decaject[Title/Abstract])) OR (Decaject-L.A.[Title/Abstract])) OR (Decaject L.A.[Title/Abstract])) OR (Hexadrol[Title/Abstract])  #8 glucocorticosteroid[Title/Abstract]  #9 corticosteroid[Title/Abstract]  #10 methylprednisone[Title/Abstract]  #11 batamethasone[Title/Abstract]  #12 #1 OR #2 OR #3 OR #4 OR #5 OR #6 OR #7 OR #8 OR #9 OR #10 OR #11  #13 ((((((((((((((((((((((((((((((("Arthroplasty, Replacement, Knee"[Mesh]) OR (Arthroplasties, Replacement, Knee[Title/Abstract])) OR (Arthroplasty, Knee Replacement[Title/Abstract])) OR (Knee Replacement Arthroplasties[Title/Abstract])) OR (Knee Replacement Arthroplasty[Title/Abstract])) OR (Replacement Arthroplasties, Knee[Title/Abstract])) OR (Knee Arthroplasty, Total[Title/Abstract])) OR (Arthroplasty, Total Knee[Title/Abstract])) OR (Total Knee Arthroplasty[Title/Abstract])) OR (Replacement, Total Knee[Title/Abstract])) OR (Total Knee Replacement[Title/Abstract])) OR (Knee Replacement, Total[Title/Abstract])) OR (Knee Arthroplasty[Title/Abstract])) OR (Arthroplasty, Knee[Title/Abstract])) OR (Arthroplasties, Knee Replacement[Title/Abstract])) OR (Replacement Arthroplasty, Knee[Title/Abstract])) OR (Arthroplasty, Replacement, Partial Knee[Title/Abstract])) OR (Unicompartmental Knee Arthroplasty[Title/Abstract])) OR (Arthroplasty, Unicompartmental Knee[Title/Abstract])) OR (Knee Arthroplasty, Unicompartmental[Title/Abstract])) OR (Unicondylar Knee Arthroplasty[Title/Abstract])) OR (Arthroplasty, Unicondylar Knee[Title/Abstract])) OR (Knee Arthroplasty, Unicondylar[Title/Abstract])) OR (Partial Knee Arthroplasty[Title/Abstract])) OR (Arthroplasty, Partial Knee[Title/Abstract])) OR (Knee Arthroplasty, Partial[Title/Abstract])) OR (Unicondylar Knee Replacement[Title/Abstract])) OR (Knee Replacement, Unicondylar[Title/Abstract])) OR (Partial Knee Replacement[Title/Abstract])) OR (Knee Replacement, Partial[Title/Abstract])) OR (Unicompartmental Knee Replacement[Title/Abstract])) OR (Knee Replacement, Unicompartmental[Title/Abstract])  #14 #12 AND #13 |
| The Cochrane search terms were as follows:147  ID Search  #1 MeSH descriptor: [Glucocorticoids] explode all trees  #2 MeSH descriptor: [Hydrocortisone] explode all trees  #3 MeSH descriptor: [Cortisone] explode all trees  #4 MeSH descriptor: [Prednisone] explode all trees  #5 MeSH descriptor: [Prednisolone explode all trees  #6 MeSH descriptor: [Triamcinolone] explode all trees  #7 MeSH descriptor: [Dexamethasone] explode all trees  #8 glucocorticosteroid: ti,ab,kw  #9 corticosteroid: ti,ab,kw  #10 methylprednisone: ti,ab,kw  #11 batamethasone: ti,ab,kw  #12 #1 OR #2 OR #3 OR #4 OR #5 OR #6 OR #7 OR #8 OR #9 OR #10 OR #11  #13 MeSH descriptor: [Arthroplasty, Replacement, Knee] explode all trees  #14 #12 AND #13 |
| The EMBASE search terms were as follows:203  #1 'Glucocorticoids':ab,ti OR 'Hydrocortisone':ab,ti OR 'Cortisone':ab,ti OR 'Prednisone':ab,ti OR 'Prednisolone':ab,ti OR 'Triamcinolone':ab,ti OR 'Dexamethasone':ab,ti  #2 knee arthroplasty:ab,ti  #3 #1 AND #2 |
| The Web of Science search terms were as follows:109  #1 TS=(Glucocorticoids OR Glucocorticoid OR Glucocorticoid Effect OR Effect, Glucocorticoid OR Glucorticoid Effects OR Effects, Glucorticoid)  #2 TS=(Hydrocortisone OR Cortisol OR Hydrocortisone, (9 beta,10 alpha,11 alpha)-Isomer OR Hydrocortisone, (11 alpha)-Isomer OR 11-Epicortisol OR 11 Epicortisol OR Epicortisol OR Cortifair OR Cortril)  #3 TS=(Cortisone OR Cortisone Acetate OR 17-Hydroxy-3,11,20-trioxopregn-4-en-21-yl acetate OR Cortone Acetate OR Adreson)  #4 TS=(Prednisone OR DehydrocDehydrocortisoneortisone OR delta-Cortisone OR Rectodelt OR Prednison Hexal OR Sterapred OR Ultracorten OR Winpred OR Apo-Prednisone OR Cortan OR Cortancyl OR Panafcort OR Cutason OR Decortin OR Dacortin OR Decortisyl OR Deltasone OR Encortone OR Encorton OR Enkortolon OR Kortancyl OR Liquid Pred OR Meticorten OR Orasone OR Panasol OR Predni Tablinen OR Prednidib OR Predniment OR Prednison Acsis OR Acsis, Prednison OR Pronisone OR Sone OR Prednison Galen)  #5 TS=(Prednisolone OR Predate OR Predonine OR Di-Adreson-F OR Di Adreson F)  #6 TS=(Triamcinolone OR Volon OR Aristocort)  #7 TS=(Dexamethasone OR Methylfluorprednisolone OR Hexadecadrol OR Decameth OR Decaspray OR Dexasone OR Dexpak OR Maxidex OR Millicorten OR Oradexon OR Decaject OR Decaject-L.A. OR Decaject L.A. OR Hexadrol)  #8 TS=(glucocorticosteroid)  #9 TS=(corticosteroid)  #10 TS=(methylprednisone)  #11 TS=(batamethasone)  #12 #1 OR #2 OR #3 OR #4 OR #5 OR #6 OR #7 OR #8 OR #9 OR #10 OR #11  #13 TS=(Arthroplasty, Replacement, Knee OR Arthroplasties, Replacement, Knee OR Arthroplasty, Knee Replacement OR Knee Replacement Arthroplasties OR Knee Replacement Arthroplasty OR Replacement Arthroplasties, Knee OR Knee Arthroplasty, Total OR Arthroplasty, Total Knee OR Total Knee Arthroplasty OR Replacement, Total Knee OR Total Knee Replacement OR Knee Replacement, Total OR Knee Arthroplasty OR Arthroplasty, Knee OR Arthroplasties, Knee Replacement OR Replacement Arthroplasty, Knee OR Arthroplasty, Replacement, Partial Knee OR Unicompartmental Knee Arthroplasty OR Arthroplasty, Unicompartmental Knee OR Knee Arthroplasty, Unicompartmental OR Unicondylar Knee Arthroplasty OR Arthroplasty, Unicondylar Knee OR Knee Arthroplasty, Unicondylar OR Partial Knee Arthroplasty OR Arthroplasty, Partial Knee OR Knee Arthroplasty, Partial OR Unicondylar Knee Replacement OR Knee Replacement, Unicondylar OR Partial Knee Replacement OR Knee Replacement, Partial OR Unicompartmental Knee Replacement OR Knee Replacement, Unicompartmental)  #14 #12 AND #13 |
