# Supplementary material for: Efficacy of additional corticosteroids to multimodal cocktail periarticular injection in total knee arthroplasty: a meta-analysis of randomized controlled trials
Source: J Orthop Surg Res. 2021 Jan 22;16:77. doi: 10.1186/s13018-020-02144-0 (PMC7821531; doi:10.1186/s13018-020-02144-0)
Supplement: Supplementary file 2 — Additional file 2: Supplementary Table 2. Raw data. [file 13018_2020_2144_MOESM2_ESM.docx]

Supplementary Table 2 Raw data

VAS score at rest (operation night)

| Study | Corticosteroids group | | | Control group | | |
| --- | --- | --- | --- | --- | --- | --- |
|  | MD | SD | Total | MD | SD | Total |
| Kim 2015 | 2.8 | 2.4 | 43 | 3.2 | 2.5 | 43 |
| Kwon 2014 | 1.2 | 2.4 | 76 | 2.3 | 3.3 | 76 |
| Seah 2011 | 1.5 | 1.4 | 50 | 1.8 | 3 | 50 |
| Takuya 2019 | 0.66 | 1.15 | 21 | 0.89 | 1.48 | 20 |
| Tsukada 2016 | 0.6 | 1.5 | 35 | 0.5 | 1.5 | 36 |
| Yue 2013 | 4 | 3 | 36 | 4.4 | 3.2 | 36 |

VAS score at rest (POD1)

| Study | Corticosteroids group | | | Control group | | |
| --- | --- | --- | --- | --- | --- | --- |
|  | MD | SD | Total | MD | SD | Total |
| Christensen 2009 | 4.8 | 2.1 | 39 | 4.1 | 3.1 | 37 |
| Ikeuchi 2013 | 1.42 | 1.25 | 20 | 4.28 | 1.83 | 20 |
| Kim 2015 | 3.4 | 2.6 | 43 | 3.6 | 3.2 | 43 |
| Kwon 2014 | 3.2 | 2 | 76 | 3.8 | 3 | 76 |
| Seah 2011 | 2.55 | 1.5 | 50 | 2.2 | 1.1 | 50 |
| Takuya 2019 | 1.35 | 1.91 | 21 | 3.52 | 2.57 | 20 |
| Tsukada 2016 | 1.7 | 1.75 | 38 | 2.6 | 2 | 36 |
| Wang 2020 | 3.4 | 0.7 | 52 | 3.6 | 0.8 | 50 |
| Yue 2013 | 6.1 | 4.4 | 36 | 6.4 | 3.6 | 36 |

VAS score at rest (POD2)

| Study | Corticosteroids group | | | Control group | | |
| --- | --- | --- | --- | --- | --- | --- |
|  | MD | SD | Total | MD | SD | Total |
| Kim 2015 | 3.1 | 3 | 43 | 3.3 | 2.9 | 43 |
| Kwon 2014 | 2.9 | 2.2 | 76 | 3.5 | 3 | 76 |
| Seah 2011 | 1.34 | 0.5 | 50 | 1.72 | 0.7 | 50 |
| Takuya 2019 | 2.07 | 2.21 | 21 | 3.36 | 2.3 | 20 |
| Tsukada 2016 | 3.4 | 2.3 | 38 | 3.9 | 2.75 | 36 |
| Wang 2020 | 2.8 | 0.6 | 52 | 3 | 0.8 | 50 |
| Yue 2013 | 3.4 | 3 | 36 | 3.6 | 3.3 | 36 |

VAS score at rest (POD3)

| Study | Corticosteroids group | | | Control group | | |
| --- | --- | --- | --- | --- | --- | --- |
|  | MD | SD | Total | MD | SD | Total |
| Christensen 2009 | 2.2 | 2.1 | 39 | 2.1 | 2.2 | 37 |
| Ikeuchi 2013 | 2.73 | 1.66 | 20 | 4.31 | 1.84 | 20 |
| Kim 2015 | 3 | 2.1 | 43 | 3.1 | 2.5 | 43 |
| Kwon 2014 | 3.5 | 2.1 | 76 | 4.1 | 3 | 76 |
| Seah 2011 | 0.81 | 1 | 50 | 1.1 | 1.4 | 50 |
| Takuya 2019 | 1.51 | 1.55 | 21 | 2.76 | 1.78 | 20 |
| Tsukada 2016 | 2.9 | 1.5 | 35 | 3 | 2 | 35 |
| Yue 2013 | 2.5 | 3 | 36 | 2.5 | 2.1 | 36 |

VAS score at rest (POD4)

| Study | Corticosteroids group | | | Control group | | |
| --- | --- | --- | --- | --- | --- | --- |
|  | MD | SD | Total | MD | SD | Total |
| Seah 2011 | 0.78 | 1.23 | 50 | 1.03 | 1.35 | 50 |
| Takuya 2019 | 1.18 | 1.39 | 21 | 2.04 | 2.2 | 20 |

VAS score at rest (POD5)

| Study | Corticosteroids group | | | Control group | | |
| --- | --- | --- | --- | --- | --- | --- |
|  | MD | SD | Total | MD | SD | Total |
| Seah 2011 | 0.77 | 1.32 | 50 | 1.1 | 1.53 | 50 |
| Takuya 2019 | 1.35 | 1.68 | 21 | 2.04 | 1.81 | 20 |

VAS score at rest (POD7)

| Study | Corticosteroids group | | | Control group | | |
| --- | --- | --- | --- | --- | --- | --- |
|  | MD | SD | Total | MD | SD | Total |
| Ikeuchi 2013 | 2.84 | 1.81 | 20 | 3.25 | 1.46 | 20 |
| Kwon 2014 | 3.2 | 2.37 | 76 | 3.7 | 2.56 | 76 |

VAS score at rest (2W)

| Study | Corticosteroids group | | | Control group | | |
| --- | --- | --- | --- | --- | --- | --- |
|  | MD | SD | Total | MD | SD | Total |
| Chia 2013 | 0.65 | 0.29 | 40 | 0.75 | 0.31 | 39 |
| Ikeuchi 2013 | 2.12 | 1.79 | 20 | 2.28 | 1.23 | 20 |
| Kwon 2014 | 3.2 | 3 | 76 | 3.7 | 2.5 | 76 |

VAS score on motion (POD1)

| Study | Corticosteroids group | | | Control group | | |
| --- | --- | --- | --- | --- | --- | --- |
|  | MD | SD | Total | MD | SD | Total |
| Takuya 2019 | 2.4 | 2.2 | 21 | 4.4 | 2.7 | 20 |
| Tsukada 2016 | 3.4 | 2 | 38 | 4.9 | 2 | 37 |
| Wang 2020 | 6.1 | 1 | 52 | 5.6 | 1 | 50 |
| Yue 2013 | 8.3 | 3.7 | 36 | 8.2 | 4.5 | 36 |

VAS score on motion (POD2)

| Study | Corticosteroids group | | | Control group | | |
| --- | --- | --- | --- | --- | --- | --- |
|  | MD | SD | Total | MD | SD | Total |
| Takuya 2019 | 2.7 | 2.3 | 21 | 3.6 | 1.9 | 20 |
| Tsukada 2016 | 4.6 | 2 | 38 | 5.1 | 2.5 | 37 |
| Wang 2020 | 4.8 | 1.3 | 52 | 4.7 | 0.9 | 50 |
| Yue 2013 | 5 | 4 | 36 | 5 | 4.5 | 36 |

VAS score on motion (POD3)

| Study | Corticosteroids group | | | Control group | | |
| --- | --- | --- | --- | --- | --- | --- |
|  | MD | SD | Total | MD | SD | Total |
| Takuya 2019 | 1.9 | 2.2 | 21 | 3.4 | 1.9 | 20 |
| Tsukada 2016 | 4 | 1.75 | 38 | 4.2 | 1.75 | 37 |
| Yue 2013 | 4.1 | 3 | 36 | 4.3 | 2.8 | 36 |

Range of flexion motion (POD1)

| Study | Corticosteroids group | | | Control group | | |
| --- | --- | --- | --- | --- | --- | --- |
|  | MD | SD | Total | MD | SD | Total |
| Christensen 2009 | 78.1 | 13.7 | 39 | 73.7 | 15.1 | 37 |
| Kim 2015 | 54 | 30 | 43 | 46 | 26 | 43 |
| Seah 2011 | 59.45 | 35 | 50 | 54.11 | 32 | 50 |
| Takuya 2019 | 71 | 13 | 21 | 71 | 13 | 20 |
| Tsukada 2016 | 67 | 11 | 38 | 57 | 18 | 37 |
| Wang 2020 | 89.1 | 11.6 | 52 | 82.3 | 18 | 50 |
| Yue 2013 | 63 | 25 | 36 | 63.5 | 21 | 36 |

Range of flexion motion (POD2)

| Study | Corticosteroids group | | | Control group | | |
| --- | --- | --- | --- | --- | --- | --- |
|  | MD | SD | Total | MD | SD | Total |
| Kim 2015 | 70 | 34 | 43 | 60 | 38 | 43 |
| Seah 2011 | 80 | 21 | 50 | 71 | 18 | 50 |
| Takuya 2019 | 77 | 10 | 21 | 80 | 8 | 20 |
| Tsukada 2016 | 73 | 15 | 38 | 65 | 18 | 37 |
| Wang 2020 | 97.6 | 8.3 | 52 | 94.1 | 14.2 | 50 |
| Yue 2013 | 82 | 32 | 36 | 80 | 27 | 36 |

Range of flexion motion (POD3)

| Study | Corticosteroids group | | | Control group | | |
| --- | --- | --- | --- | --- | --- | --- |
|  | MD | SD | Total | MD | SD | Total |
| Christensen 2009 | 91.8 | 8.4 | 39 | 89.5 | 110.8 | 37 |
| Kim 2015 | 86 | 24 | 43 | 88 | 16 | 43 |
| Seah 2011 | 96 | 24 | 50 | 83 | 21 | 50 |
| Takuya 2019 | 87 | 10 | 21 | 85 | 9 | 20 |
| Tsukada 2016 | 78 | 15 | 38 | 76 | 11 | 37 |
| Wang 2020 | 106.5 | 7.6 | 52 | 105.4 | 12.1 | 50 |
| Yue 2013 | 90 | 25 | 36 | 95 | 30 | 36 |

Range of flexion motion (POD4)

| Study | Corticosteroids group | | | Control group | | |
| --- | --- | --- | --- | --- | --- | --- |
|  | MD | SD | Total | MD | SD | Total |
| Seah 2011 | 94.6 | 12.78 | 50 | 90.54 | 13.94 | 50 |
| Takuya 2019 | 92 | 9 | 21 | 90 | 10 | 20 |
| Tsukada 2016 | 86 | 15 | 38 | 84 | 12.5 | 38 |

Range of flexion motion (POD5)

| Study | Corticosteroids group | | | Control group | | |
| --- | --- | --- | --- | --- | --- | --- |
|  | MD | SD | Total | MD | SD | Total |
| Seah 2011 | 93.79 | 15.14 | 50 | 90.8 | 13.21 | 50 |
| Takuya 2019 | 94 | 10 | 21 | 94 | 9 | 20 |
| Tsukada 2016 | 87 | 16.25 | 38 | 84 | 13.75 | 38 |

Range of flexion motion (POD7)

| Study | Corticosteroids group | | | Control group | | |
| --- | --- | --- | --- | --- | --- | --- |
|  | MD | SD | Total | MD | SD | Total |
| Kim 2015 | 121 | 14 | 43 | 119 | 20 | 43 |
| Kwon 2014 | 106.2 | 10.2 | 76 | 105.2 | 8.9 | 76 |
| Seah 2011 | 95 | 13 | 50 | 90 | 15 | 50 |
| Tsukada 2016 | 91 | 11.25 | 38 | 88 | 10 | 37 |
| Yue 2013 | 100 | 20 | 36 | 102 | 23 | 36 |

Range of flexion motion (2W)

| Study | Corticosteroids group | | | Control group | | |
| --- | --- | --- | --- | --- | --- | --- |
|  | MD | SD | Total | MD | SD | Total |
| Chia 2013 | 95.7 | 13.1 | 40 | 93.7 | 13.8 | 39 |
| Tsukada 2016 | 95 | 13.75 | 38 | 95 | 15 | 37 |

Range of flexion motion (4W)

| Study | Corticosteroids group | | | Control group | | |
| --- | --- | --- | --- | --- | --- | --- |
|  | MD | SD | Total | MD | SD | Total |
| Seah 2011 | 101.96 | 20.14 | 50 | 91.55 | 17.96 | 50 |
| Tsukada 2016 | 104 | 11.25 | 38 | 108 | 15 | 38 |

Range of flexion motion (6W)

| Study | Corticosteroids group | | | Control group | | |
| --- | --- | --- | --- | --- | --- | --- |
|  | MD | SD | Total | MD | SD | Total |
| Chia 2013 | 112.1 | 15.6 | 42 | 113.1 | 9.7 | 43 |
| Christensen 2009 | 108.5 | 12.5 | 39 | 107.7 | 15.3 | 37 |

Range of flexion motion (12W)

| Study | Corticosteroids group | | | Control group | | |
| --- | --- | --- | --- | --- | --- | --- |
|  | MD | SD | Total | MD | SD | Total |
| Chia 2013 | 119.3 | 12 | 40 | 120.2 | 7.6 | 39 |
| Christensen 2009 | 112.4 | 12.1 | 39 | 112.5 | 10.3 | 37 |
| Seah 2011 | 106.94 | 18 | 50 | 101.84 | 22 | 50 |
| Tsukada 2016 | 115 | 13.75 | 38 | 120 | 10 | 37 |
| Wang 2020 | 115.5 | 6.1 | 52 | 116 | 7.5 | 50 |

Range of flexion motion (24W)

| Study | Corticosteroids group | | | Control group | | |
| --- | --- | --- | --- | --- | --- | --- |
|  | MD | SD | Total | MD | SD | Total |
| Kwon 2014 | 131.1 | 10.7 | 76 | 128.3 | 12.5 | 76 |
| Seah 2011 | 111.1 | 20.13 | 50 | 105.59 | 17.54 | 50 |
| Tsukada 2016 | 121 | 20 | 38 | 120 | 18.75 | 38 |

Range of extension motion (POD1)

| Study | Corticosteroids group | | | Control group | | |
| --- | --- | --- | --- | --- | --- | --- |
|  | MD | SD | Total | MD | SD | Total |
| Takuya 2019 | 8 | 3 | 21 | 8 | 5 | 20 |
| Tsukada 2016 | 6 | 2.5 | 38 | 9 | 5 | 38 |

Range of extension motion (POD2)

| Study | Corticosteroids group | | | Control group | | |
| --- | --- | --- | --- | --- | --- | --- |
|  | MD | SD | Total | MD | SD | Total |
| Takuya 2019 | 8 | 3 | 21 | 9 | 5 | 20 |
| Tsukada 2016 | 7 | 3.75 | 38 | 10 | 6.25 | 38 |

Range of extension motion (POD3)

| Study | Corticosteroids group | | | Control group | | |
| --- | --- | --- | --- | --- | --- | --- |
|  | MD | SD | Total | MD | SD | Total |
| Takuya 2019 | 7 | 3 | 21 | 8 | 4 | 20 |
| Tsukada 2016 | 6 | 2.5 | 38 | 9 | 6.25 | 38 |

Range of extension motion (POD4)

| Study | Corticosteroids group | | | Control group | | |
| --- | --- | --- | --- | --- | --- | --- |
|  | MD | SD | Total | MD | SD | Total |
| Takuya 2019 | 6 | 4 | 21 | 7 | 4 | 20 |
| Tsukada 2016 | 7 | 3.75 | 38 | 8 | 5 | 38 |

Range of extension motion (POD5)

| Study | Corticosteroids group | | | Control group | | |
| --- | --- | --- | --- | --- | --- | --- |
|  | MD | SD | Total | MD | SD | Total |
| Takuya 2019 | 6 | 4 | 21 | 6 | 5 | 20 |
| Tsukada 2016 | 6 | 2 | 38 | 8 | 5 | 38 |

Morphine equivalent

| Study | Corticosteroids group | | | Control group | | |
| --- | --- | --- | --- | --- | --- | --- |
|  | MD | SD | Total | MD | SD | Total |
| Christensen 2009 | 46 | 22.4 | 39 | 47.8 | 35.1 | 36 |
| Ikeuchi 2013 | 9.16 | 19.16 | 20 | 18.33 | 25.82 | 20 |
| Seah 2011 | 10.03 | 4.12 | 50 | 15.49 | 5.36 | 50 |
| Wang 2020 | 13.1 | 4.4 | 52 | 17.2 | 4.5 | 50 |
| Yue 2013 | 23 | 21 | 36 | 23.5 | 26 | 36 |

Postoperative drainage

| Study | Corticosteroids group | | | Control group | | |
| --- | --- | --- | --- | --- | --- | --- |
|  | MD | SD | Total | MD | SD | Total |
| Ikeuchi 2013 | 346 | 201 | 20 | 423 | 210 | 20 |
| Kwon 2014 | 458.4 | 156.1 | 76 | 491.8 | 182.1 | 76 |
| Seah 2011 | 281 | 157 | 50 | 246 | 148 | 50 |

Length of hospital stay

| Study | Corticosteroids group | | | Control group | | |
| --- | --- | --- | --- | --- | --- | --- |
|  | MD | SD | Total | MD | SD | Total |
| Christensen 2009 | 2.6 | 0.7 | 39 | 3.5 | 1.9 | 37 |
| Seah 2011 | 5.2 | 1.3 | 50 | 6.8 | 2 | 50 |
| Wang 2020 | 3.17 | 0.47 | 52 | 3.12 | 0.52 | 50 |

Time for straight leg raising

| Study | Corticosteroids group | | | Control group | | |
| --- | --- | --- | --- | --- | --- | --- |
|  | MD | SD | Total | MD | SD | Total |
| Kwon 2014 | 2.26 | 0.903 | 76 | 2.88 | 0.833 | 76 |
| Seah 2011 | 2.3 | 1.1 | 50 | 2.8 | 0.9 | 50 |

Surgical site infection

| Study | Corticosteroids group | | Control group | |
| --- | --- | --- | --- | --- |
|  | Event | Total | Event | Total |
| Chia 2013 | 1 | 42 | 0 | 43 |
| Christensen 2009 | 1 | 39 | 0 | 37 |
| Ikeuchi 2013 | 0 | 20 | 0 | 20 |
| Kim 2015 | 0 | 43 | 0 | 43 |
| Kwon 2014 | 0 | 76 | 0 | 76 |
| Seah 2011 | 1 | 50 | 1 | 50 |
| Takuya 2019 | 0 | 21 | 0 | 20 |
| Tsukada 2016 | 0 | 38 | 0 | 38 |
| Wang 2020 | 0 | 52 | 0 | 50 |

Tendon rupture

| Study | Corticosteroids group | | Control group | |
| --- | --- | --- | --- | --- |
|  | Event | Total | Event | Total |
| Seah 2011 | 0 | 50 | 0 | 50 |
| Wang 2020 | 0 | 52 | 0 | 50 |

Nausea and vomiting

| Study | Corticosteroids group | | Control group | |
| --- | --- | --- | --- | --- |
|  | Event | Total | Event | Total |
| Ikeuchi 2013 | 1 | 20 | 4 | 20 |
| Kim 2015 | 24 | 43 | 21 | 43 |
| Kwon 2014 | 14 | 76 | 12 | 76 |
| Tsukada 2016 | 3 | 38 | 3 | 37 |
| Wang 2020 | 21 | 52 | 19 | 50 |

Wound ozzing

| Study | Corticosteroids group | | Control group | |
| --- | --- | --- | --- | --- |
|  | Event | Total | Event | Total |
| Kwon 2014 | 6 | 76 | 5 | 76 |
| Tsukada 2016 | 1 | 38 | 1 | 37 |
| Wang 2020 | 6 | 52 | 4 | 50 |
